# Hematological parameters to predict post-COVID-19 immune response among vaccinated and nonvaccinated individuals: a retrospective cross-sectional study

**DOI:** 10.1097/MS9.0000000000002064

**Published:** 2024-04-16

**Authors:** Qaisar A. Khan, Tahira Atta, Tamara Tango, Arif Mumtaz, Priyadharshini Saravanan, Sree H. Vallabhaneni, Ismail K. Shinwari, Bhavana Vattikuti, Rukhsar Jan, Ravina Verma, Nayab Sami, Ameer M. Farrukh, Yaxel Levin-Carrion

**Affiliations:** a Khyber Teaching Hospital MTI KTH, Peshawar; b KMU Institute of Medical Sciences Kohat; c DHQ and Teaching Hospital KDA Kohat, Pakistan; d Pondicherry Institute of Medical Sciences, Pondicherry; e Apollo Institute of Medical Science and Research, Hyderabad, India; f Faculty of Medicine Universitas, Indonesia; g Cebu Doctors’ University College of Medicine, Philippines; h St. Georges Medical University, True Blue, Grenada; i University of Galway, School of Medicine, Ireland; j Rutgers New Jersey Medical School, New Jersey, USA

**Keywords:** antibodies, COVID-19, full blood count, hematological parameters, immunity

## Abstract

**Background:**

This study finds the changes in the hematological parameters of healthy individuals to predict the immune status against coronavirus disease 2019 (COVID-19) among COVID -19 vaccinated and nonvaccinated individuals.

**Methods:**

A comparative cross-sectional study among 210 healthy individuals was conducted. All individuals were divided into three groups, that is, IgG positive, IgG negative, and IgG and IgM positive, based on ELISA. Data analysis was done using SPSS version 25 for Windows.

**Results:**

A statistically significant effect was found among the three groups in terms of mean levels of hemoglobin (Hb), hematocrit (Hct), mean corpuscular hemoglobin concentration (MCHC), red blood cells (RBC), RDW-CV, lymphocyte, neutrophil, eosinophils, and basophil count. The study also showed that 52.8% (*n*=74) had neither taken vaccination nor had any history of previous COVID-19 infection but were IgG antibody positive.

**Conclusion:**

There was a statistically significant difference among hematological parameters between immune and nonimmune groups, and it can predict the COVID-19 immune status.

## Introduction

Highlights

- This study finds the changes in the hematological parameters of healthy individuals to predict the immune status against coronavirus disease 2019 (COVID-19) and to find whether herd immunity against COVID-19 has developed in the population of Pakistan.
- There was a statistically significant difference among hematological parameters including Hb, Hct, MCHC, RBC count, RDW-CV, lymphocyte, neutrophil, eosinophil, and basophil count between immune and nonimmune groups, and can predict the COVID-19 immune status. Furthermore, the study indicated that herd immunity against COVID-19 is developing in Pakistan.
- The herd immunity against COVID-19 is in developing phase among the population of Pakistan.

Coronavirus disease 2019 (COVID-19), caused by the severe acute respiratory syndrome coronavirus-2 (SARS-CoV-2), was first identified in Wuhan, China, in December 2019, followed by the widespread of the disease globally^1^. The WHO announced the global COVID-19 pandemic on 11 March 2020^2,3^. Primarily, coronaviruses were labeled as pathogens responsible for respiratory tract infections, ranging from mild to moderate upper respiratory tract infections. However, the SARS-CoV-2 had also negatively impacted the other organ systems, such as hematological, immunological, neurological, gastrointestinal, and cardiovascular systems, resulting in severe systemic diseases^4^. COVID-19 affected a billion people within half a year, and subsequently, more than half a million people died^5^.

The COVID-19 pandemic had a devastating impact on the already overburdened healthcare system, influencing the physical, psychological, and economic survival of humans. The rapid transmission of the virus through respiratory aerosols has infused various protective behaviors in humans, both individually using personal protective equipment and socially as social distancing, quarantine of the infected patients, and massive population testing, representing crucial strategies for mitigating. However, these methods remained insufficient to end the COVID-19 pandemic^6^.

The COVID-19 vaccine has been a significant defensive line against the infection. Vaccines are one of the most efficient and cost-effective interventions against epidemics and contagious diseases. Vaccines have a proven protective role for individuals and communities^7^. Herd or community immunity is the immunization of the population at a massive level to protect immunocompromised and nonvaccinated components of society^8^. Herd immunity could be achieved actively through vaccination or passively after recovering from the infection. Immunization of 80% of the population globally against smallpox had achieved herd immunity to a level resulting in eradication of the disease^7^. For SARS-CoV-2, the resistance acquirement of 50–67% of the community is required to achieve herd immunity. The vaccination-based herd immunity had restraints at multiple levels regarding pathogen epitope stability, and there are multiple stereotypes, antigenic shift and drift, and immune imprinting. The inadequacy of healthcare policies and population immune incompetence, such as underdeveloped immunity in the young, immune senescence, and immune deficiency, also hinder herd immunity^9^.

In immunity against SARS-CoV-2, the role of B cells is vital in cytokine production, antigen presentation, and antibody secretion, including isotypes, predominantly immunoglobulin G (IgG) and immunoglobulin M (IgM). The high plasma levels of proinflammatory cytokines, including interleukins and tumor necrosis factor-α, had been reported in SARS-CoV-2-affected patients^10^. Vaccination coverage may be used to achieve herd immunity levels; however, vaccinated individuals could remain susceptible to disease, and unvaccinated individuals could be immune even without vaccination^11^. Monitoring the immunity of the population to COVID-19 could help identify communities at risk of outbreak and determine community-targeted vaccination efforts. The reverse transcription-polymerase chain reaction (RT-PCR), enzyme-linked immunosorbent assays (ELISA), hybridization microarray assays, and high-resolution computed tomography (HRCT) of chest are diagnostic investigations for COVID-19, requiring highly developed laboratory infrastructure, specialized professionals and long reporting time, and are not cost-effective for lower-middle-income countries (LMICs)^12^.

Hematological and immunological markers had been possible predictors of COVID-19 immune response and prognosis of the disease. Complete blood count and coagulation profile had a prominent predicting role in COVID-19^13^. The neutrophil-to-lymphocyte ratio (NLR), platelet-to-lymphocyte ratio (PLR), and lymphocyte-to-monocyte ratio (LMR) are immune markers being used for prediction in viral pneumonia. NLR is a well-known prognostic marker and has an independent association with mortality in the general population and various diseases as sepsis, pneumonia, COVID-19, cardiovascular disorders, malignancies, and postsurgical complications^14^. NLR had a substantial diagnostic and prognostic role in COVID-19, attributed to its being a cost-effective, rapid, and readily available prognostic indicator^15^.

Understanding the immunological status of noninfected or nonvaccinated individuals had significance in assessing the community’s herd immunity level. There is a significant correlation between the hematological parameters to predict the severity and outcome of disease. To evaluate the association of hematological parameters with immunological factors in noninfected individuals, it is also required to indicate the status of immunity against COVID-19. This study aims to determine the changes in hematological parameters of noninfected individuals to predict the immunity status against COVID-19. It intends to discover the current status of herd immunity against COVID-19 in Pakistan’s population.

## Materials and Methods

### Study design

A comparative retrospective cross-sectional study was conducted from July to November 2022.

### Sample size

WHO sample size calculator was used for sample size calculation. A total of 210 healthy individuals participated in the study, out of which 140 participants were immune to COVID-19, 64 were nonimmune, that is, participants whose IgG and IgM were negative for COVID-19, and 6 were asymptomatic, that is, participants whose IgM was positive for COVID-19 but were asymptomatic.

### Data collection and management

The participants’ demographic information includes age, sex, vaccination status, data about the previous COVID-19 infection, family history, current or past systemic diseases, and drug history. Each individual is then tested for COVID-19 antibodies through SARS-COV-2IGM/IGG AB TEST FANEL (WB/9/P) REF; H100 then confirmed by ELISA. ELISA test was performed with a fully automated Chemiluminescence Immunoassay Analyzer (Abbot Architect Ci8200) using FDA-authorized reagent kits (Elbascience SARS-Cov ELISA Kit). Based on ELISA, the participants were classified into three groups: IgG positive, IgG negative, and IgG and IgM positive. Participants with IgG levels of >1.4 were classified as immune to COVID-19 infection. A complete blood count test was done using a CBC analyzer (Sysmex hematology analyzer kx-21) for all the participants in each group, and a single CBC analyzer then did it. Various hematological parameters were then compared according to cut-off values decided for these hematological parameters^16^. The work has been reported in line with the strengthening the reporting of cohort, cross-sectional, and case–control studies in surgery (STROCSS) guidelines^17^.

### Inclusion and exclusion criteria

Only healthy individuals aged 20 to 60 years were included in the study. Those with active infection, systemic disorders, hematological malignancies, anemias, or autoimmune conditions were excluded from the analysis. Individuals who smoked or used any medication routinely or recreationally and those who donated blood or had a history of blood transfusions in the last 3 months were also excluded from the study. No restrictions of sex, occupation, or education were applied to conduct this study.

### Data analysis

Statistical analysis used the One-way ANOVA test to compare numerical data that were normally distributed and that involved more than two unpaired groups. The distribution of the data was analyzed using SPSS version 25. Kolmogorov–Smirnov was used for the number of samples greater than or equal to 50, while Shapiro–Wilk was used for the number of samples less than 50.

## Results

A total of 210 patients were included in this study, with a mean age of 32.48±14.63 years. Overall 54.3% were males (*n*=114) and 45.7% were females (*n*=96). Among immune participants, 59.7% were male and 40.3% were female, while in nonimmune and those with active COVID-19 infection 42.18%, 50% were male, and 57.8%, and 50% were female, respectively. Among these 210 patients, 66.7% were only immunoglobulin G (IgG) positive (*n*=140), 30.5% were antibody negative (*n*=64), and 2.8% were positive for IgG and immunoglobulin M (IgM). Regarding vaccination history, about 47.1% of patients in the only IgG-positive group had been vaccinated, and most had been vaccinated within one year. In addition, 39% of patients in the antibody-negative group had been vaccinated within less than a year (*n*=3), within a year (*n*=5), more than a year ago (*n*=4), and unknown date of vaccination (*n*=13). No patients in the IgG and IgM-positive groups had been vaccinated previously. The vaccines used during vaccination in the only IgG-positive group were varied, dominated by Sinovac (92.4%), followed by Pfizer (3.0%), Sinopharm, Moderna, and Sputnik. Sinovac vaccinated all the patients in the antibody-negative group. 95.2% of patients had not acquired the COVID-19 infection. Of those who had COVID-19 infections, nine of them were in the IgG-positive group, while only one patient was from the antibody-negative group. A summary of participant’s demographic information is given in Table 1.

**Table 1:** Participant’s demographic information and vaccination history

	Only IgG Positive	IgG Negative	Both IgG and IgM Positive
Number of patients	140	64	6
Male	84	27	3
Female	56	37	3
Mean age (range)	33.5 (20–60)	30.1 (20–60)	34.7 (27–56)
Currently vaccinated			0
• Less than 1 year	1	3	
• 1 year back	65	5	
• More than 1 year back	0	4	
• Vaccination date unknown	0	13	
No vaccination done	74	39	6
Sinovac	61	25	0
Sinopharm	1	0	0
Moderna	1	0	0
Pfizer	2	0	0
Sputnik	1	0	0
Previous COVID-19 infection			
<1 year	1	1	0
1 year back	7	0	0
2 years back	1	0	0
No history of previous COVID-19 infection	131	63	6

### Hematological parameters

Variation of hematological parameters in participants among three groups based on their immune status is given in Table 2.

**Table 2:** Variations in hematological parameters among the immunological groups

Mean hematological parameters	Cut off values	Only IgG Positive	Antibody Negative	IgG and IgM Positive	*P*
Hemoglobin (g/dl)		14.4±1.8	13.5±1.5	12.9±1.3	0.027
Male	11.5–17.3				
Female	11–16				
Hematocrit (%)		41.1±5.1	38.6±4.7	39.8±3.3	0.004
Male	34–53.90				
Female	36–46				
MCV (fl)	84–98	79.7±8.1	77.8±7.4	84.4±4.7	0.076
MCH (pg)	27.5–32.4	28.0±3.6	26.8±3.7	27.4±1.9	0.111
MCHC (g/dl)	31.7–34.2	35.0±2.3	34.9±2.0	32.4±0.6	0.018
RBC (cells/mm^3^)	(4.50 – 5.50) ×10^6^	(5.20±0.7) × 10^6^	(5.00±0.6) × 10^6^	(4.7±0.3) × 10^6^	0.046
RDW-CV (%)	11.1–14	15.1±2.1	15.8±2.2	13.7±0.9	0.015
Leukocyte (/mm^3^)	5–11×10^3^	(12.9±44.7) × 10^3^	(8.1±2.4) × 10^3^	(9.9±3.3) × 10^3^	0.686
Neutrophil (%)	40–60%	60.0±8.9	56.4±8.1	58.5±8.3	0.023
Lymphocyte (%)	1.19–40	33.8±8.6	37.3±8.3	36.8±7.4	0.025
Monocyte (%)	2–8%	2.5±1.1	4.0±0.7	3.2±1.2	0.001
Eosinophil (%)	1–4%	3.5±2.0	2.0±0.5	1.5±0.5	0.001
Basophil (%)	0.5–1%	1.5±1.9	0.0±0.0	0.0±0.0	0.001
Platelet (cells/mm^3^)	150–450×10^3^	267 364.3±74 104.1	256 703.1±60 179.5	266 666.7±94 326.4	0.606
NLR	1–3	2.1±1.6	1.6±0.5	1.7±0.6	0.080

Cells/mm^2^, cells per square millimeter; fL, femtoliter; g/dl, grams per deciliter; MCH, mean corpuscular hemoglobin; MCHC, mean corpuscular hemoglobin concentration; MCV, mean corpuscular volume; NLR, neutrophil-lymphocyte ratio; pg, picogram; RBC, red blood cell; RDW-CV, red cell distribution width.

The details of hematological parameters in all three immunological groups are described as follows:

#### Hemoglobin (Hb)

There was a significant effect of mean Hb at *P*<0.05 for three immunological groups [F (2, 207)=7.85, *P*=0.027]. The Post-hoc Tamhane test indicated that the mean score for Hb level in the IgG-positive group was significantly different from than the antibody-negative group (M=0.59, SE=0.56, *P*=0.706) in terms of statistics (M=0.92, SE=0.25, *P*=0.001). However, the mean Hb level in the IgG-positive group (M=1.51, SE=0.55, *P*=0.100) and antibody-negative group (M=0.59, SE=0.56, *P*=0.706) did not significantly differ from IgG and IgM-positive groups.

#### Hematocrit (Hct)

A significant effect of mean Hct was seen among three immunological groups [F(2, 207)=5.70, *P*=0.004]. The Post-hoc Tamhane test for the mean hematocrit level in the IgG-positive group significantly differed from the mean Hct level in the antibody-negative group (M=2.51, SE=0.73, *P*=0.002). Mean Hct level was not different statistically in the IgG-positive group (M=1.34, SE=1.41, *P*=0.757) and antibody-negative group (M=1.2, SE=1.46, *P*=0.835) did not significantly differ from IgG and IgM-positive groups.

#### Mean corpuscular volume (MCV)

There were no significant effects of MCV among the IgG-positive, antibody-negative, and IgM and IgG-positive groups [F(2, 207)=2.61, *P*=0.076].

#### Mean corpuscular hemoglobin (MCH)

There was no significant effect of MCH among the three immunological groups [F (2, 207)=2.22, *P*=0.111].

#### Mean corpuscular hemoglobin concentration (MCHC)

There was a significant effect of MCHC among the IgG-positive, antibody-negative, and IgG and IgM-positive groups [F (2, 207)=4.10, *P*=0.018]. The Post-hoc Tamhane test demonstrated that the mean score MCHC level in the IgG-positive group was significantly different from those in the IgG and IgM-positive groups in terms of statistics (M=2.61, SE=0.30, *P*=0.000). Moreover, a significant difference in mean corpuscular hemoglobin concentration was also seen between the antibody-negative and IgM and IgG-positive groups (M=2.52, SE=0.34, *P*=0.000).

#### Red blood cells (RBCs)

A significant effect of mean RBCs was observed among the IgG-positive, antibody-negative, and IgM and IgG-positive groups [F (2, 207)=3.12, *P*=0.046]. The Post-hoc Tamhane test for mean RBCs in the IgG-positive group significantly differed from those in IgM and IgG-positive groups (M=0.49, SE=0.24, *P*=0.028).

#### Red cell distribution width (RDW-CV)

There was a significant effect of RDW-CV among the three immunological groups [F (2, 207)=4.3, *P*=0.015]. The Post-hoc Tamhane test for the mean RDW-CV in the IgG-positive group was significantly different from the mean RDW-CV in the IgM and IgG-positive groups (M=1.34, SE=0.39, *P*=0.028). In addition, a significant difference in RDW-CV was also seen between the antibody-negative and IgM and IgG-positive groups (M=2.06, SE=0.44, *P*=0.002).

#### Total leukocyte count (TLC)

There was no significant effect of mean TLC among the IgG-positive group, antibody-negative, and IgM and IgG-positive groups [F(2, 207)=0.378, *P*=0.686].

#### Neutrophil count

A significant effect of mean neutrophil count among the three immunological groups [F(2, 207)=3.83, *P*=0.023]. At least two groups had significant differences in terms of mean neutrophil count (*P*=0.023). The Post-hoc Tamhane test demonstrated a significant difference in mean neutrophil count between the IgG-positive group and negative antibody group (*P*=0.015). However, it was not clinically significant (mean difference=3.6).

#### Lymphocyte percentage

There was a significant effect of mean lymphocyte percentage among the IgG-positive, antibody-negative, and IgM and IgG-positive groups [F (2, 207=3.76, *P*=0.025]. Also, there were at least two groups with significant differences in mean lymphocyte percentage (*P*=0.025). According to the Post-hoc Tamhane result, there was a significant difference in mean lymphocyte percentage between the IgG-positive and antibody-negative group (*P*=0.023) with no difference clinically (mean difference=3.44).

#### Monocyte count

There was a significant effect of mean monocyte count among the IgG-positive, antibody-negative, and IgM and IgG-positive groups [F (2, 207)=47.83, *P*=0.000]. At least two groups had a significant difference in mean monocyte count in terms of statistics (*P*=0.000). The Post-hoc Tamhane test showed a statistically significant difference in mean monocyte count among the IgG-positive and antibody-negative groups (*P*=0.000). However, it was not clinically significant (mean difference=1.43).

#### Eosinophil count

A significant effect of mean eosinophil count was observed among the three groups [F (2, 207)=20.25, *P*=0.000]. Post-hoc Tamhane test demonstrated a statistically significant difference in mean eosinophil count between the IgG and antibody-negative group (*P*=0.000) as well as between the IgG-positive and IgM and IgG-positive groups (*P*=0.000) having a mean difference of 1.51 and 1.99, respectively.

#### Basophil count

There was a significant effect of mean basophil count among the IgG-positive, antibody-negative, and IgM and IgG-positive groups [F (2, 207)=21.17, *P*=0.000]. The Post-hoc Tamhane test showed a significant difference in mean basophil between the IgG-positive and IgM and IgG group (*P*=0.000). However, the difference was not clinically significant (mean difference=1.52).

#### Platelet count

There was no significant effect of mean platelet count among the three immunological groups [F (2, 207)=0.50, *P*=0.606].

#### NLR

There was no significant effect of mean NLR among the three immunological groups [F (2, 207)=2.56, *P*=0.080].

#### Immunity status against COVID-19

Furthermore, out of 140 IgG-positive participants, 47% (*n*=74) were vaccinated, 1% (*n*=1) had a history of COVID-19-infection, and 52% (*n*=73) were neither vaccinated but were still IgG positive and were immune to COVID-19, indicating the development of immunity against COVID-19 in the population of Pakistan (Table 3).

**Table 3:** Vaccination status in comparison to previous infection (n=140)

Variables	History of COVID-19 infection		
IgG positive		Without previous infection	Previous infection
Vaccinated	66 (47%)	58 (87%)	12 (8%)
Non vaccinated	74 (52%)	73 (98%)	1 (1%)

## Discussion

The novel scope of this research included investigating various hematological parameters to predict immune response in individuals not infected with COVID-19. The hematological parameters include Hb, Hct, MCV, MCHC, WBCs, and differential leukocyte count.

The statistically significant difference in Hb level was observed among the IgG positive and antibody negative groups, being higher in the immune group and indicating the impact of immunological status on Hb level. Hb level had been considered a prognostic indicator for the severity of COVID-19. A low level of Hb on presentation was associated with poor disease outcomes and demanded early risk stratification and medical intervention^18^. This low Hb level could be related to the action of the virus against the development or degradation of RBC or the existence of comorbidities^14^. A statistically significant Hct level difference was found between IgG-positive and antibody-negative groups. Jalil *et al*.^19^ reported a statistically significant association of Hct level among COVID-19 patients and noninfected individuals (*P*–value<0.001).

The statistically significant differences in RDW-CV had been observed between IgG-positive and IgG and IgM-positive groups and antibody-negative and IgG and IgM-positive groups, indicating the potential impact of immunological responses on the size of RBCs. Sarkar *et al*. reported that the higher level of RDW in COVID-19 patients was associated with poor outcomes. Critically ill and deceased COVID-19 patients have higher levels of RDW on admission. The level of RDW higher than 14.5 had an association with a higher risk of mortality^20^.

A statistically significant difference in neutrophil count, lymphocyte percentage, and monocyte count was found between IgG-positive and antibody-negative groups. Dubey *et al*. had reported a substantial difference of neutrophil (*P*=0.002), lymphocyte (*P*=0.004), and monocyte (*P*=0.003) between mild and moderate cases of COVID-19 and a significant difference of neutrophil (*P*=0.000) and lymphocyte (*P*=0.000) between moderate and severe cases^21^, the IgG-positive group, had higher levels of mean neutrophil count. Neutrophils are the primary responders to tissue injury and are increased in the initial 4 h of trauma, contributing to tissue repair. Neutrophilia had been considered a poor prognostic indicator for the severity of COVID-19. Lymphocytes, such as B, T, and natural killer (NK) cells, had an essential role against viral infections. Lymphopenia, neutrophilia, and thrombocytopenia are typical responses to COVID-19 infection. However, the persistence of lymphopenia for a long duration had an association with severity and poor prognosis. Monocytes had an essential role in inflammatory responses through antigen presentation and phagocytosis. The rapid decrease in monocyte count in the initial 3 days of trauma, followed by a rapid rise during 5–7 days, indicates a poor prognosis. The gradual decline in the initial 5 days of tissue injury followed by a slight increase in 5–14 days means a good prognosis^22^.

TLC and NLR had no statistically significant difference among all three immunological groups. Contradictorily, Tooriet *et al*.^23^ also found elevated neutrophil-to-lymphocyte ratio as a valuable predictor for severity and mortality of SARS-CoV-2 infection. Leukocytosis, neutrophilia, and increased neutrophil to lymphocyte ratio (NLR), possibly due to inflammatory response, are significantly associated with the disease severity. NLR was highest in patients with critical disease^24^. However, these hematological parameters had no significant clinical variance as they were in the range of normal values. Moreover, for each parameter, there is a specific life span that could change with factors such as smoking, alcohol, dehydration, systemic illnesses, and various medications.

Literature regarding the hematological markers as indicators of immunity needs to be improved. Hematological parameters contribute to disease monitoring after detecting diseases by variable values. Moreover, a combination of hematological characteristics can be used to estimate severity or outcome. Hematological parameters cannot directly predict the post-COVID-19 immune response, although estimates could be done to indicate the presence of immunity against COVID-19. These parameters are variable, and further research is needed to estimate the ratios among various parameters that would be constant and individual-specific.

Fifty-two percent of the IgG-positive participants had neither been vaccinated with the COVID-19 vaccine nor had a history of previous COVID-19 infection, indicating the development of immunity against COVID-19 among the population of Pakistan. Herd immunity is an essential concept for epidemic control^25^. It can be acquired in two ways, either by infection with the pathogen or via vaccination. Using safe and effective vaccines inducing herd immunity reduces disease prevalence and decreases mortality and morbidity^26^. This type of immunity is necessary, but it presents several difficulties because various social and economic variables can delay it, and vaccine hesitancy may threaten communities to develop herd immunity^27^. Pakistan had faced a significant obstacle, mostly in terms of vaccine hesitancy, a major roadblock to eradicating vaccine-preventable diseases. However, the awareness of vaccines is one of the most prominent reasons for vaccination among the general population^28^. In Pakistan, the COVID-19 vaccine has been introduced gradually to immunize people to achieve herd immunity^29^. Five vaccines (AstraZeneca, Sinopharm, CanSino, Sputnik V, and Gamaleya) and the phased rollout method of mass immunization had been approved as of 29 June 2021^30^. Currently, the healthcare system of Pakistan has not been subjected to the worst effects of the pandemic, but large vaccination campaigns, with an estimated 84% of Pakistan’s population aged 12 or older receiving all recommended vaccinations, helping the community to achieve the immunity against COVID-19^31^.

### Limitations

The study results cannot apply to the population with active infection, immune deficiency, using medications such as steroids, antiplatelet, anticoagulants, antiepileptics, etc., and family history of hematological disorders or systemic diseases. The small sample size is another limitation of this study; further research with a large sample size is needed.

## Conclusion

The study concluded that some hematological parameters in the complete blood picture were statistically different in immune people than in nonimmune. These parameters include Hb, Hct, MCHC, RBC count, RDW-CV, lymphocyte, neutrophil, eosinophil, and basophil count that can predict immunity against COVID-19. However, these parameters are similar because the study has been conducted on clinically healthy individuals. Further research is needed to find out the ratios of these hematological parameters, which are individual, that can further support this hypothesis. Furthermore, it has been concluded from the study that immunity against COVID-19 is developing in Pakistan.

## Ethical approval

Ethical approval was obtained from DHQ and Teaching Hospital Kohat Ethical Review Board on 22 May 2022 with reference number 3223.

## Consent

Written informed consent was obtained from all participants before recruiting in this study.

## Sources of funding

No funding was received for conducting this study.

## Author contribution

Q.A.K., T.A., A.M., R.J., and I.K.S.: conceptualized the study and were responsible for the data collection; Q.A.K., T.T., and P.S.: were responsible for data analysis; S.H.V., B.V., A.M.F., R.V., and Y.L.C.: wrote the original manuscript; Q.A.K., T.A., A.M., and N.S.: critically revised the manuscript and did the final editing. All the co-authors reviewed and approved the manuscript before submission.

## Conflicts of interest disclosure

There is no conflicts of interest to declare.

## Research registration unique identifying number (UIN)

1. Name of the registry: Research Registry.
2. Unique identifying number or registration ID: researchregistry9812.
3. Browse the Registry - https://www.researchregistry.com/browse-theregistry#home/?view_2_search=9812&view_2_page=1&view_2_sort=field_2|desc.

## Guarantor

Dr Qaisar Ali Khan.

## Data availability statement

Data can be available upon reasonable request to the corresponding author.

## Provenance and peer review

Not commissioned, externally peer- reviewed.

## References

[1] Sharma A Tiwari S Deb MK . Severe acute respiratory syndrome coronavirus-2 (SARS-CoV-2): a global pandemic and treatment strategies. Int J Antimicrob Agents 2020;56:106054. 32534188 10.1016/j.ijantimicag.2020.106054 PMC7286265

[2] Cucinotta D Vanelli M . WHO declares COVID-19 a pandemic. Acta Bio Medica: Atenei Parmensis 2020;91:157. 10.23750/abm.v91i1.9397 PMC7569573 32191675

[3] Hasan MN Haider N Stigler FL . The global case-fatality rate of COVID-19 has been declining since May 2020. Am J Trop Med Hyg 2021;104:2176. 33882025 10.4269/ajtmh.20-1496 PMC8176487

[4] World Health Organization . Transmission of SARS-CoV-2: implications for infection prevention precautions: scientific brief 09 July 2020. World Health Organization; 2020.

[5] Tahir MJ Saqlain M Tariq W . Population preferences and attitudes towards COVID-19 vaccination: a cross-sectional study from Pakistan. BMC Public Health 2021;21:1–12. 34565351 10.1186/s12889-021-11814-5 PMC8474768

[6] Khalid A Ali S . COVID-19 and its challenges for the healthcare system in Pakistan. Asian Bioethics Rev 2020;12:551–564. 10.1007/s41649-020-00139-x PMC7424236 32837562

[7] Frederiksen LSF Zhang Y Foged C . The long road toward COVID-19 herd immunity: vaccine platform technologies and mass immunization strategies. Front Immunol 2020;11:1817. 32793245 10.3389/fimmu.2020.01817 PMC7385234

[8] Neagu M . The bumpy road to achieve herd immunity in COVID-19. J Immunoassay Immunochem 2020;41:928–945. 33086932 10.1080/15321819.2020.1833919

[9] Mallory ML Lindesmith LC Baric RS . Vaccination-induced herd immunity: successes and challenges. J All Clin Immunol 2018;142:64–66. 10.1016/j.jaci.2018.05.007 PMC6433118 29803799

[10] Chen S Guan F Candotti F . The role of B cells in COVID-19 infection and vaccination. Front Immunol 2022;13:988536. 36110861 10.3389/fimmu.2022.988536 PMC9468879

[11] Fisman DN Amoako A Tuite AR . Impact of population mixing between vaccinated and unvaccinated subpopulations on infectious disease dynamics: implications for SARS-CoV-2 transmission. CMAJ 2022;194:E573–E580. 35470204 10.1503/cmaj.212105 PMC9054088

[12] Sheikhzadeh E Eissa S Ismail A . Diagnostic techniques for COVID-19 and new developments. Talanta 2020;220:121392. 32928412 10.1016/j.talanta.2020.121392 PMC7358765

[13] Al‐Saadi EAKD Abdulnabi MA . Hematological changes associated with COVID‐19 infection. J Clin Lab Anal 2022;36:e24064. 34783405 10.1002/jcla.24064 PMC8646489

[14] Buonacera A Stancanelli B Colaci M . Neutrophil to lymphocyte ratio: an emerging marker of the relationships between the immune system and diseases. Int J Mol Sci 2022;23:3636. 35408994 10.3390/ijms23073636 PMC8998851

[15] Keski H . Hematological and inflammatory parameters to predict the prognosis in COVID-19. Ind J Hematol Blood Transf 2021;37:534–542. 10.1007/s12288-021-01407-y PMC7910775 33679013

[16] Bates I . 2 - Reference Ranges and Normal Values. In: Bain BJ, Bates I, Laffan MA editors. Dacie and Lewis Practical Haematology, 12th edn. Elsevier; 2017:8–17.

[17] Mathew G Agha R for the STROCSS Group . STROCSS 2021: strengthening the reporting of cohort, cross-sectional and case-control studies in surgery. Int J Surg 2021;96:106165. 34774726 10.1016/j.ijsu.2021.106165

[18] Algassim AA Elghazaly AA Alnahdi AS . Prognostic significance of hemoglobin level and autoimmune hemolytic anemia in SARS-CoV-2 infection. Ann Hematol 2021;100:37–43. 32918594 10.1007/s00277-020-04256-3 PMC7486165

[19] Jalil AT Shanshool MT Dilfy SH . Hematological and serological parameters for detection of COVID-19. J Microbiol Biotechnol Food Sci 2022;11:e4229–e4229.

[20] Sarkar S Kannan S Khanna P . Role of red blood cell distribution width, as a prognostic indicator in COVID‐19: a systematic review and meta‐analysis. Rev Med Virol 2022;32:e2264. 34091982 10.1002/rmv.2264 PMC8209859

[21] Dubey DB Mishra S Reddy HD . Hematological and serum biochemistry parameters as a prognostic indicator of severally ill versus mild Covid-19 patients: a study from tertiary hospital in North India. Clin Epidemiol Global Health 2021;12:100806. 10.1016/j.cegh.2021.100806 PMC8214724 34179566

[22] Fouladseresht H Ghamar Talepoor A Eskandari N . Potential immune indicators for predicting the prognosis of COVID-19 and trauma: similarities and disparities. Front Immunol 2022;12:785946. 35126355 10.3389/fimmu.2021.785946 PMC8815083

[23] Toori KU Qureshi MA Chaudhry A . Neutrophil to lymphocyte ratio (NLR) in COVID-19: a cheap prognostic marker in a resource constraint setting. Pak J Med Sci 2021;37:1435. 34475926 10.12669/pjms.37.5.4194 PMC8377926

[24] Elkhalifa AM Elderdery AY Al Bataj IA . Hematological findings among COVID-19 patients attending King Khalid Hospital at Najran, Kingdom of Saudi Arabia. Biomed Res Int 2022;2022. 10.1155/2022/4620037 PMC8865990 35224093

[25] Desai AN Majumder MS . What is herd immunity? JAMA 2020;324:2113. 33074287 10.1001/jama.2020.20895

[26] Omer SB Yildirim I Forman HP . Herd immunity and implications for SARS-CoV-2 control. JAMA 2020;324:2095–2096. 33074293 10.1001/jama.2020.20892

[27] Suryawanshi YN Biswas DA . Herd immunity to fight against COVID-19: a narrative review. Cureus 2023;15. 10.7759/cureus.33575 PMC9909126 36779140

[28] Rasheed A Idrees W Ali Khan Q . An insight into the acceptance and hesitancy of COVID-19 vaccines in Pakistan: a cross-sectional survey. Cureus 2022;14. 10.7759/cureus.32363 PMC9826719 36628039

[29] Ali I Hamid S . Implications of COVID-19 and “super floods” for routine vaccination in Pakistan: the reemergence of vaccine preventable-diseases such as polio and measles. Hum Vaccin Immunother 2022;18:2154099. 36573023 10.1080/21645515.2022.2154099 PMC9891673

[30] Qamar MA Irfan O Dhillon RA . Acceptance of COVID-19 vaccine in Pakistan: a nationwide cross-sectional study. Cureus 2021;13. 10.7759/cureus.16603 PMC8378420 34430184

[31] Mehmood Q Ullah I Hasan MM . COVID-19 vaccine hesitancy: Pakistan struggles to vaccinate its way out of the pandemic. Ther Adv Vaccines Immunother 2022;10:25151355221077658. 35174312 10.1177/25151355221077658 PMC8841903

